# Cordycepin and a preparation from *Cordyceps militaris* inhibit malignant transformation and proliferation by decreasing EGFR and IL-17RA signaling in a murine oral cancer model

**DOI:** 10.18632/oncotarget.21477

**Published:** 2017-10-04

**Authors:** Peng-Yang Hsu, Yueh-Hsin Lin, Erh-Ling Yeh, Hui-Chen Lo, Tai-Hao Hsu, Che-Chun Su

**Affiliations:** ^1^ Department of Bioindustry Technology, Da-Yeh University, Datsuen, Taiwan; ^2^ Department of Nutrition and Dietetics, Changhua Christian Hospital, Taiwan; ^3^ Department of Nutritional Science, Fu Jen Catholic University, Taiwan; ^4^ Graduate Institute of Statistics and Information Science, National Changhua University of Education, Taiwan; ^5^ Department of Internal Medicine, Changhua Christian Hospital, Taiwan; ^6^ Laboratory of Immunology, Changhua Christian Hospital, Taiwan

**Keywords:** Cordyceps militaris, cordycepin, oral cancer, EGFR, IL-17A tumoricidal activity

## Abstract

*Cordyceps militaris* (CM) and its active ingredient cordycepin have been reported to inhibit tumor growth, but the mechanisms are not fully understood. This study used a mouse model for oral cancer and a cell line, 4NAOC-1 derived from the model to study the mechanisms. Our results show that a CM preparation (CMP) can significantly inhibit tumor development and malignant transformation in the model. *In vitro* data indicate that CMP and cordycepin can inhibit 4NAOC-1 cell proliferation, either anchorage-dependent or -independent. Cordycepin can also increase cell apoptosis, and decrease cell mitosis and EGFR signaling. In accordance, CMP treatment can significantly decrease the levels of ki-67 and EGFR signaling molecules in cancer tissues. We also found that the levels of IL-17A in cancer tissues of control mice were significantly increased, and CMP inhibited these levels. IL-17A can stimulate cancer cell proliferation, which can be suppressed by cordycepin. Furthermore, cordycepin can reduce the expression of IL-17RA and its downstream signaling molecules. Moreover, CMP and cordycepin can significantly decrease IL-17A production *in vitro* and *in vivo*. Finally, CMP and its ingredients can enhance tumoricidal activities with increase in IFN-γ and TNFα, and decrease PD-L1 expression. In conclusion, CMP and its ingredient cordycepin can inhibit tumor growth and malignant transformation in a mouse model for oral cancer via inhibition of EGFR- and IL-17RA-signaling and enhancement of anti-tumor immunity.

## INTRODUCTION

Cancer has been an important, if not the most important issue in human health since the last century. Despite witnessing huge progress, it remains a great challenge.

It is known that cancer tissues are infiltrated with immune cells, which can play either a pro-tumor or anti-tumor role [1, 2]. IL-17A is a cytokine produced by immune cells to promote tumor growth via up-regulation of IL-6, IL-8, G-CSF, VEGF, MMP-2, MMP-9, and p-STAT3 expression in cancer cells and tissues [3–11]. On the other hand, cytokines like IFN-γ and TNFα are known to regulate cancer cell apoptosis and tumor regression [8, 12–14]. Cancer immunotherapy is developed with a hope that by manipulation of the body’s own immune system, cancer can be inhibited or even cured. One example that has brought patients and doctors a great hope is the immune checkpoint inhibitors; however, for the majority of cancer patients, a durable efficacy is still not achievable [15]. Nevertheless, it still reminds us that regulation of immune responses is a promising therapeutic strategy for cancer patients.

For every type of cancer, the best prognosis always comes from those patients who are diagnosed at an early stage, and when surgery can successfully remove tumor tissues from the body. This fact reminds us that intervention should start as early as possible. When cancer cells are well grown and established in many locations, the war is definitely harder to fight [10]. Therefore, early intervention with preventive agents is important.

Ancient Chinese spared no efforts to find solutions for maintenance of health and achievement of longevity. Among the well-known remedies are Dong-Chong-Xia-Cao and *Cordyceps militaris* (CM). Both belong to the division of Ascomycete, and have been reported to have many functions, like anti-inflammation [16], anti-angiogenesis [17], anti-tumor [18, 19], and immunomodulation [20–22]. Cordycepin is one of the active ingredients in CM [19], which can cause apoptosis and cell cycle arrest through decreasing expression of Wnt [23], mTOR [24], and Erk1/2 [25] signaling pathway-related proteins. Despite many studies have demonstrated, the detailed mechanisms of CM and cordycepin are still not well understood.

Here, we used an established murine oral cancer model [26], which has many features similar to those in human oral cancer [26–29]. In particular, both benign and malignant lesions are present in a single mouse tongue. We used this model and a cell line derived from the model to study the anti-tumor mechanisms of CMP and cordycepin.

## RESULTS

### CMP inhibited tumor growth

**Figure 1:**
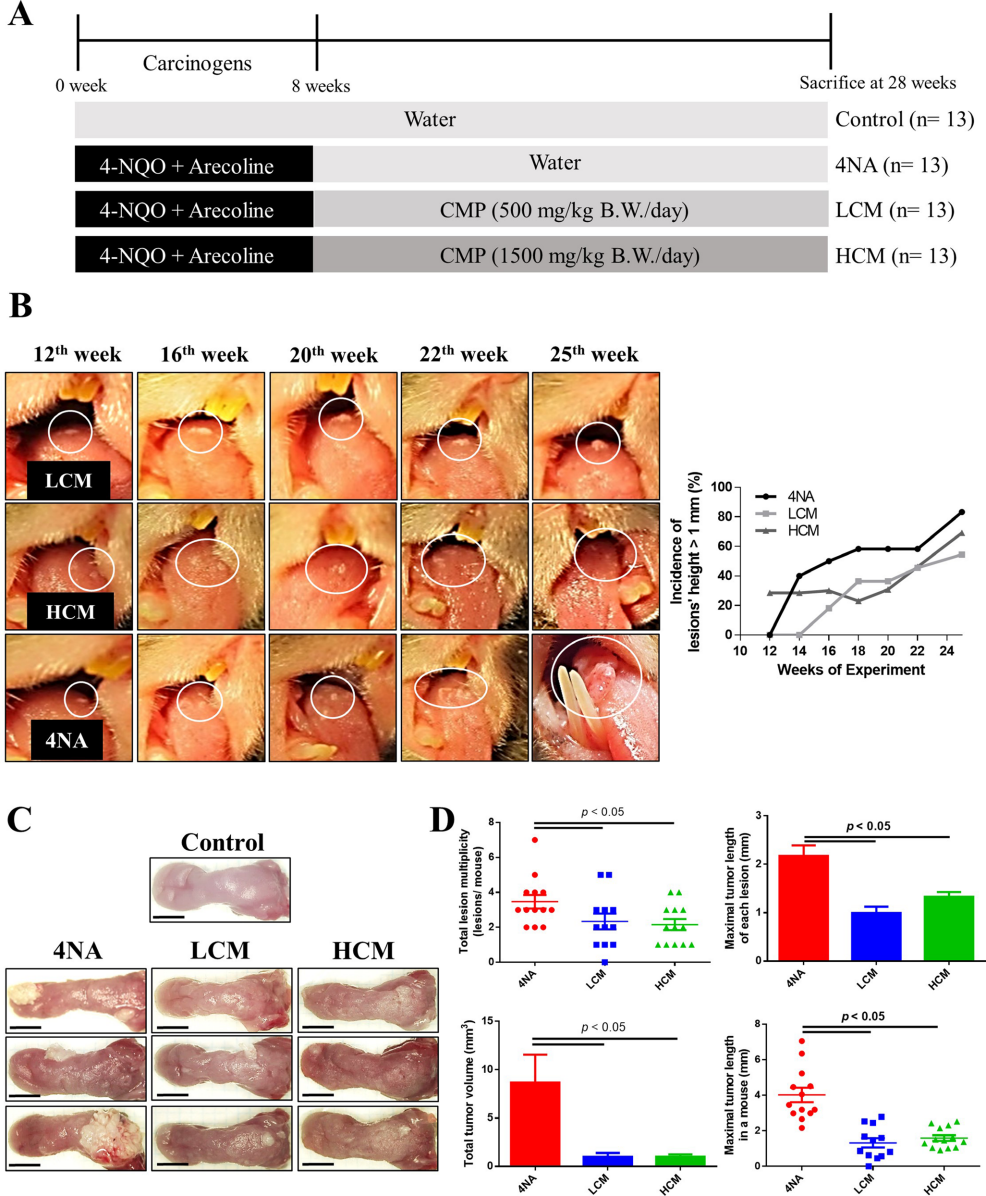
CMP inhibited tumor growth and malignant transformation in the tongue (**A**) Schematic experiment protocol in this study. 4-NQO: 4-nitroquinoline-1-oxide. CMP: *Cordyceps militaris* preparation. (**B**) Representative pictures and the incidence of oral lesions development in mice during the experimental period. (**C**) Representative tongue pictures at sacrifice. Scale bar = 3 mm. (**D**) Quantification of number, length, and volume of oral lesions in mice. Each group contained 13 mice and each data point denoted an individual mouse. Data are presented as mean ± SEM and statistical analysis is performed with one-way ANOVA following Tukey’s test.

We used a HPLC system to analyze active compounds in the CMP, and results are shown in supplementary Figure 1. Preliminary results showed that CMP at 200 mg/kg/day (*n* = 12) or 500 mg/kg/day (*n* = 12) for 20 weeks had mild to moderate inhibiting effects on oral lesions, and these mice tolerated the doses well, as judged by body weight changes (data not shown). In this study, we gave mice CMP at doses of 500 mg/kg/day (LCM group) or 1500 mg/kg/day (HCM group) for 20 weeks after exposure to oral carcinogens. The experimental protocol is illustrated in Figure 1A. Mice tolerated these doses well, as judged by body weight gains (data not shown). CMP treatment markedly inhibited development of oral lesions in mice (Figure 1B). At sacrifice, CMP treatment significantly suppressed levels of tumor volume and tumor multiplicity in the tongue, compared with those from 4NA group (Figure 1C and 1D).

### CMP inhibited malignant transformation

**Figure 2:**
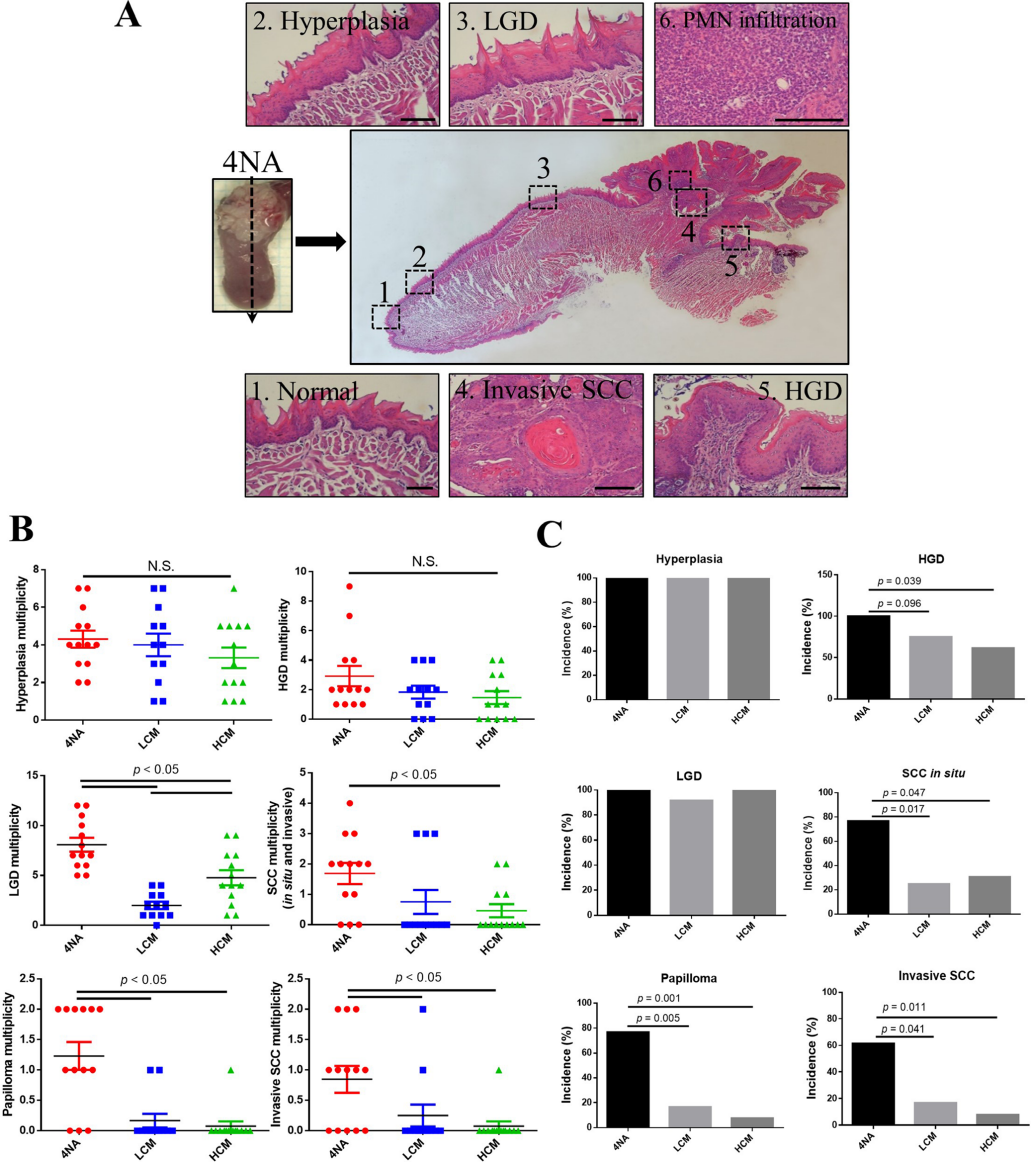
CMP inhibited malignant transformation in the tongue (**A**) Representative panel pictures of histopathological changes in a tongue section from 4NA group. Scale bar = 100 μm. PMN: polymorphonuclear cells. LDG: low-grade dysplasia. HGD: high-grade dysplasia. SCC: squamous cell carcinoma. (**B**) Multiplicity of different histopathological changes in the tongue. Each group contained 13 mice and each data point denoted an individual mouse. Data are presented as mean ± SEM and statistical analysis is effectuated with one-way ANOVA following Tukey’s test. Quantification of multiplicity is described in the Materials and Methods. N.S.: not significant. (**C**) Incidence of different histopathological changes in the tongue. Each group contained 13 mice and statistical analysis was performed with the Fisher’s exact test by SPSS software.

One characteristic of the mouse model is that lesions from benign to malignant are present in the tongue. A representative longitudinal section is shown (Figure 2A). CMP significantly suppressed the incidence and multiplicity of SCC *in situ* and invasive SCC in the tongue (Figure 2B and 2C). We also found that invasive SCC lesions in the 4NA group were intensely infiltrated with neutrophils (sub-Figure 6 of Figure 2A), a phenomenon was not observed in mice with CMP treatment (data not shown).

### Both CMP and cordycepin suppressed proliferation of 4NAOC-1 cells

**Figure 3:**
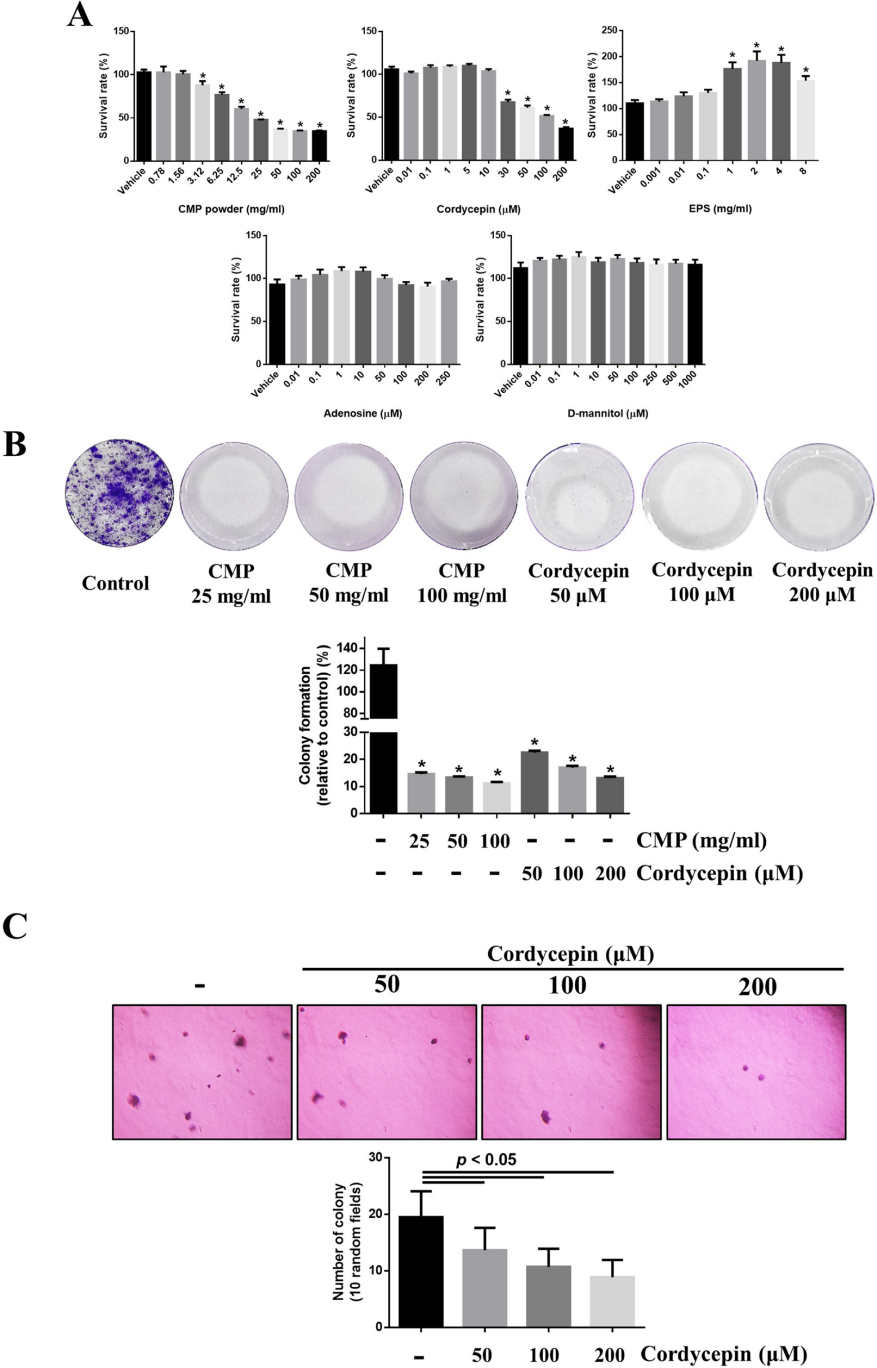
Both CMP and cordycepin suppressed proliferation of 4NAOC-1 cells (**A**) Survival of 4NAOC-1 cells after 48 hours treatment with different ingredients. Cell survival was measured by the alamar blue agent and results were shown as percentage to vehicle control. Data are presented as mean ± SD and statistical analysis is effectuated with one-way ANOVA following Tukey’s test. (**B**) Representative culture wells and quantification of colony formation. Data are presented as percentage relative to control and mean ± SD. Statistical analysis was performed with one-way ANOVA following Tukey’s test. (**C**) Representative pictures and quantification of soft agar formation assay. Data are presented as mean ± SD and statistical analysis is carried out with one-way ANOVA following Tukey’s test.

We assayed the anti-proliferation activity of these four compounds on 4NAOC-1 cells, a cell line derived from an oral cancer tissue of 4NA group. Both CMP and cordycepin significantly inhibited cell proliferation, but EPS, adenosine, and D-mannitol did not (Figure 3A). CMP or cordycepin also significantly inhibited colony formation *in vitro* (Figure 3B). Moreover, we used a soft agar assay to show that cordycepin was also potent in inhibiting cell proliferation in an anchorage-independent environment (Figure 3C).

### Both CMP and cordycepin inhibited mitosis and EGFR signaling in 4NAOC-1 cells

**Figure 4:**
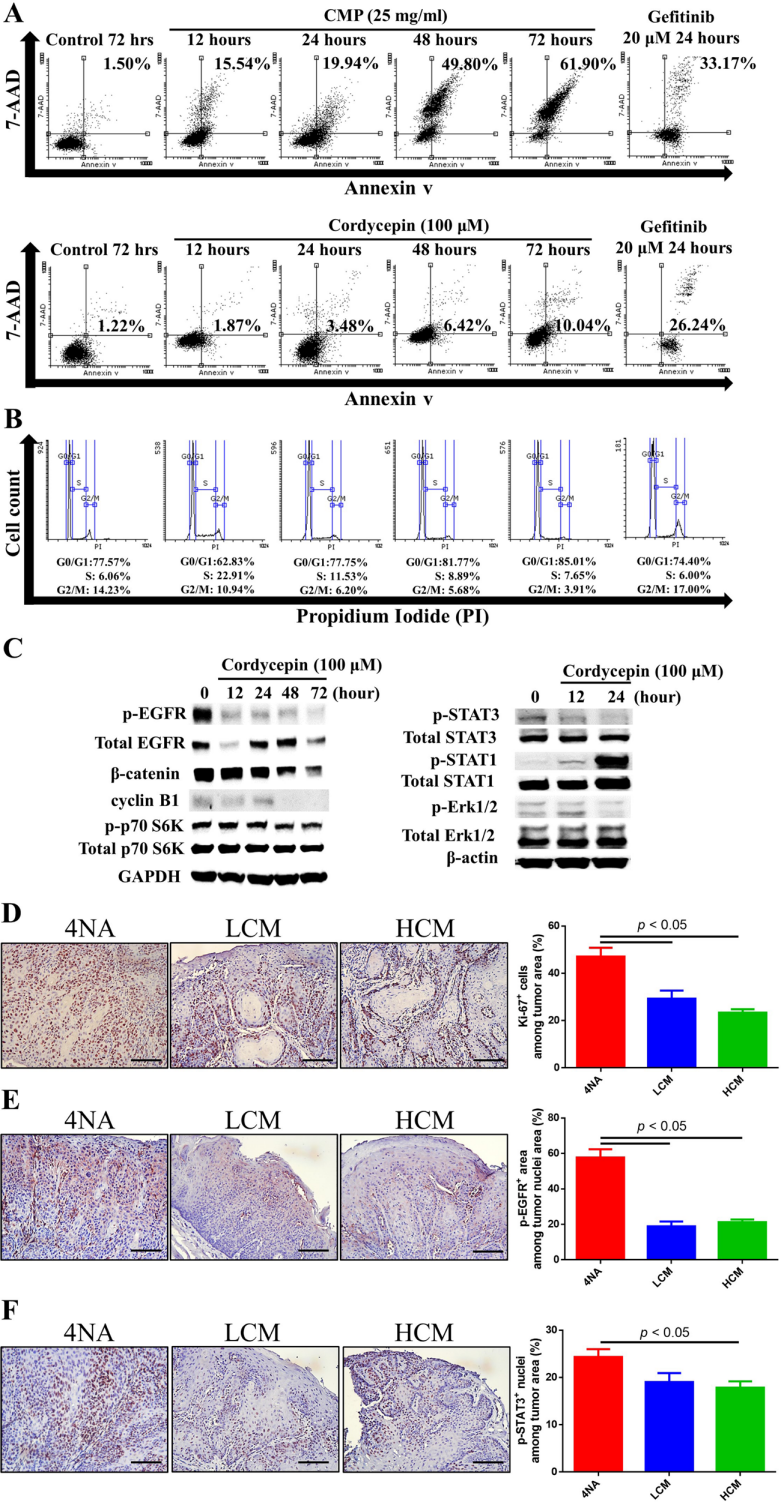
Both CMP and cordycepin inhibited mitosis and EGFR signaling (**A**) Apoptosis of 4NAOC-1 cells with CMP and cordycepin treatment. Representative results are shown. (**B**) Cell cycle analysis of 4NAOC-1 cells with cordycepin treatment. Representative results are shown. (**C**) Western blot analysis of different markers in 4NAOC-1 cells with cordycepin treatment. Representative results are shown. (**D, E,** and **F**) Representative immunohistochemical analysis and quantification of ki-67, p-EGFR (Y1068), and p-STAT3 (Y705) in squamous cell carcinoma tissues are shown. Data are presented as mean ± SEM and statistical analysis is performed with one-way ANOVA following Tukey’s test.

Cordycepin and CMP increased cell apoptosis time-dependently, though the levels at 24 hour post-treatment were less than that by gefitinib, an EGFR-specific inhibitor (Figure 4A). In addition, cordycepin treatment decreased cell cycle of G2/M phase (Figure 4B). Western blots showed that cordycepin markedly decreased expression of p-EGFR, p-Erk1/2, p-STAT3, p-p70 S6K, β-catenin, and cyclin B1, and increased expression of p-STAT1 in 4NAOC-1 cells (Figure 4C). Moreover, CMP treatment in mice significantly reduced the expression levels of ki-67, p-EGFR, and p-STAT3 in cancer tissues (Figure 4D, 4E, and 4F). Collectively, these results support our hypothesis that both CMP and cordycepin inhibit cancer cells mitosis and EGFR signaling.

### Both CMP and cordycepin inhibited IL-17RA signaling

**Figure 5:**
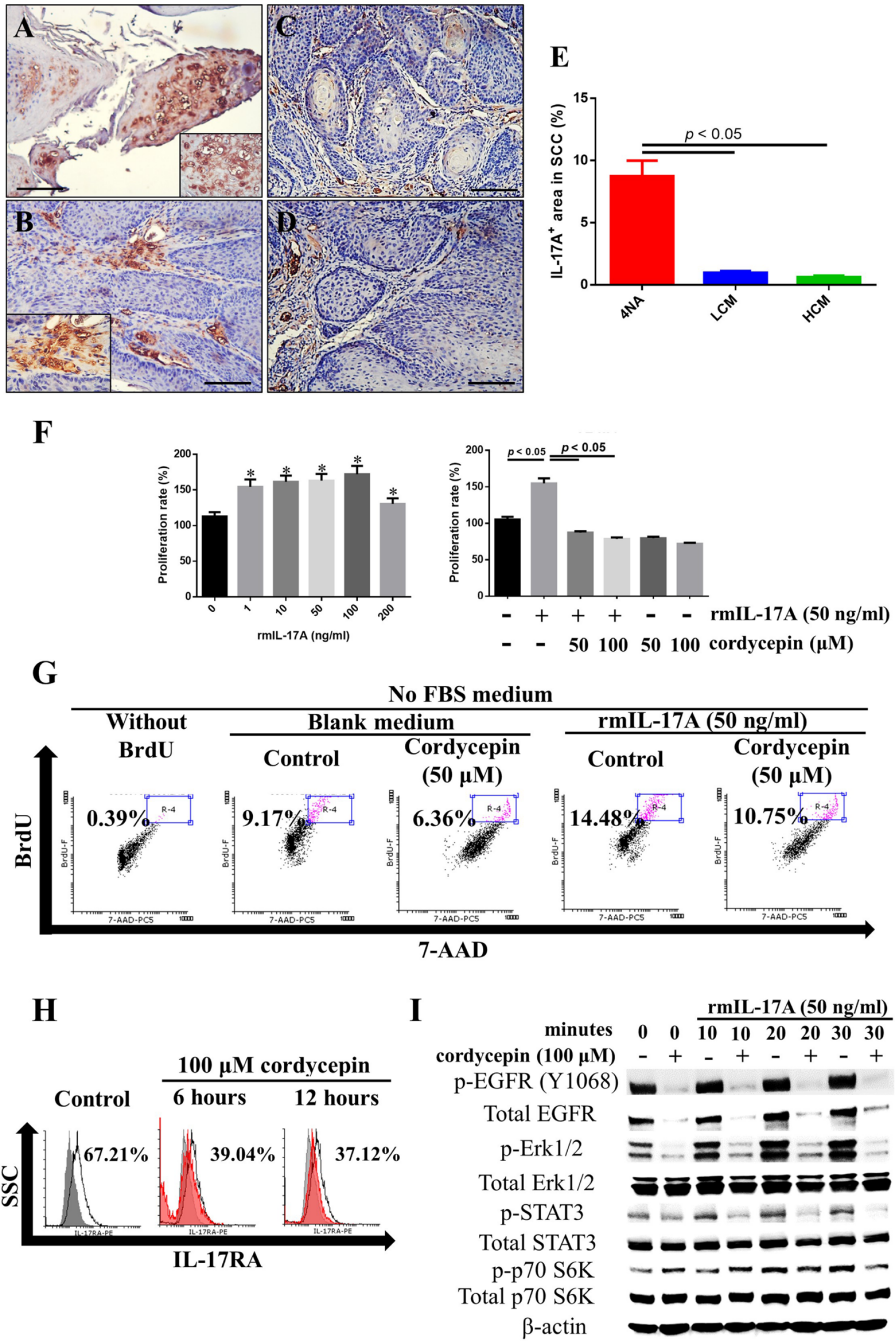
Both CMP and cordycepin inhibit IL-17A effects (**A** and **B**) Representative immunohistochemical staining of IL-17A in invasive squamous cell carcinoma (SCC) tissues of 4NA group are shown. Inserted pictures are of a higher magnification. (**C** and **D**) Immunoreactivity of IL-17A in invasive SCC tissues from LCM (C) and HCM (D) groups. Scale bar = 100 μm. (**E**) Quantification of IL-17A levels in SCC tissues of each group. Data are presented as mean ± SEM and statistical analysis is conducted with one-way ANOVA following Tukey’s test. (**F**) Recombinant IL-17A treatment for 72 hours stimulated proliferation of 4NAOC-1 cells, and cordycepin partially inhibited the proliferation. Data are presented as mean ± SD and statistical analysis is conducted with one-way ANOVA following Tukey’s test. Representative data from three independent experiments are shown. (**G**) Recombinant IL-17A treatment for 72 hours stimulated DNA synthesis in 4NAOC-1 cells and cordycepin partially inhibited the effect. Representative results are shown. BrdU (bromodeoxyuridine) was added at the last 24 hours of cultivation. (**H**) Cordycepin treatment for 6 and 12 hours decreased IL-17RA expression in 4NAOC-1 cells. Representative data are shown. (**I**) Cordycepin pre-treatment for 12 hours inhibited IL-17A/ IL-17RA signaling in 4NAOC-1 cells. Representative data from three independent tests are shown.

IL-17A was intensely expressed in the intra-tumor and peri-tumor areas of cancer tissues in 4NA group (Figure 5A and 5B), and the expression was reduced by CMP treatment (Figure 5C, 5D, and 5E). In addition, we showed that IL-17A enhanced cancer cell proliferation, which was significantly inhibited by cordycepin (Figure 5F). Moreover, IL-17A increased DNA synthesis in cancer cells, and cordycepin inhibited the effect, too (Figure 5G). As shown in Figure 5H, cordycepin significantly decreased IL-17RA expression in 4NAOC-1 cells. Moreover, cordycepin pre-treatment effectively inhibited the expression of p-Erk1/2, p-STAT3, and p-p70 S6K in 4NAOC-1 cells stimulated with IL-17A (Figure 5I). These findings all demonstrate that both CMP and cordycepin can inhibit IL-17A signaling.

### Both CMP and cordycepin reduced IL-17A production

**Figure 6:**
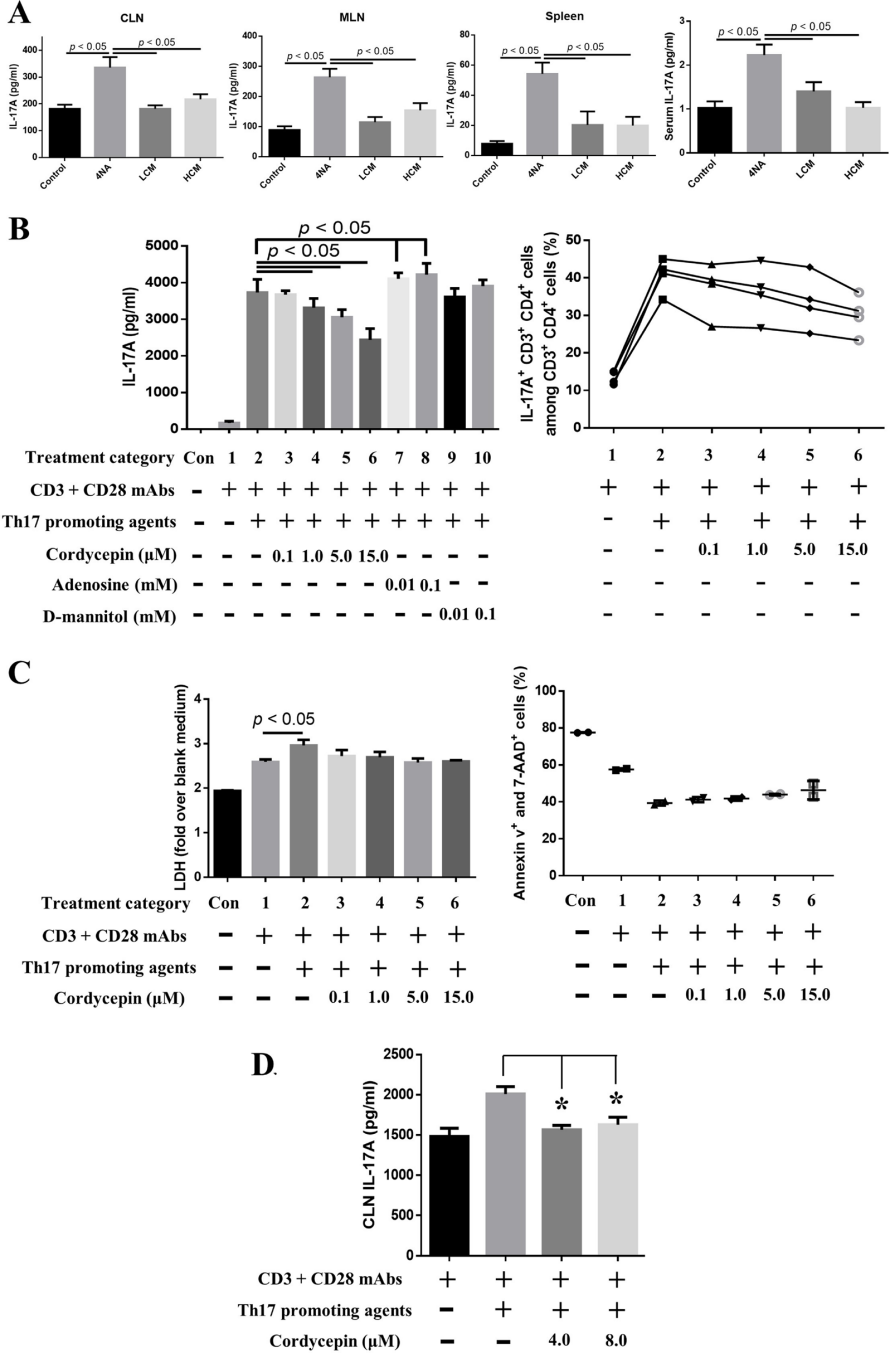
Both CMP and cordycepin inhibited the production of IL-17A (**A**) Levels of IL-17A in the culture supernatants of lymph nodes and splenocytes and in the serum. Data are presented as mean ± SEM and statistical analysis is performed with one-way ANOVA following Tukey’s test. (**B**) Cordycepin significantly inhibited IL-17A expression and IL-17A-expressing CD3^+^ CD4^+^ splenocytes after 3 days of *ex vivo* differentiation. The content of Th17 promoting agents is described in the Materials and Methods. (**C**) Cordycepin did not cause significant cell death in terms of LDH release and annexin v signaling. Data are presented as mean ± SD and statistical analysis is effectuated with one-way ANOVA following the Tukey’s test. (**D**) Cordycepin suppressed IL-17A production in tumor-draining lymph node cells. Representative results from three independent experiments are shown. Data are presented as mean ± SD and statistical analysis is conducted with one-way ANOVA following Tukey’s test. CLN: cervical lymph nodes; LDH: lactate dehydrogenase.

Levels of IL-17A in the culture supernatant of CLN, MLN, and splenocytes and in the serum were significantly increased in 4NA group, compared with LCM and HCM groups (Figure 6A). As shown in Figure 6B, cordycepin significantly inhibited IL-17A levels in the culture supernatant of splenocytes with Th17 differentiation agents in a dose-dependent manner, but adenosine and D-mannitol did not. Cordycepin also decreased the percentage of IL-17A-expressing CD3^+^ CD4^+^ cells in splenocytes (Figure 6B). The inhibition did not depend on cell death, as indicated by results from different assays (Figure 6C). Moreover, cordycepin significantly suppressed IL-17A levels in the culture supernatant of tumor-draining lymph node cells (Figure 6D). These results indicate that cordycepin can effectively inhibit IL-17A production.

### CMP and its ingredients enhanced anti-tumor immune activities

**Figure 7:**
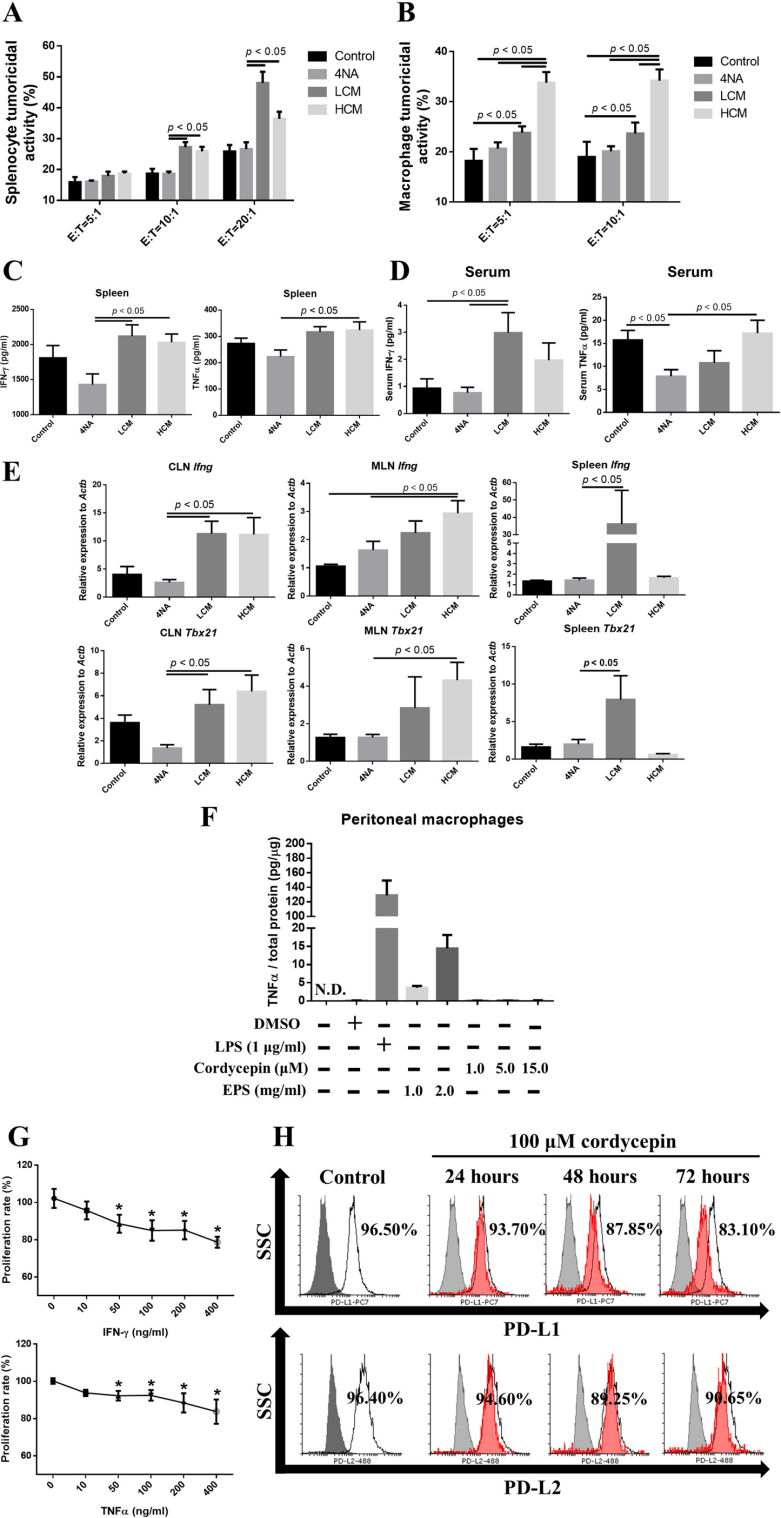
CMP and its ingredient enhanced anti-tumor immunity (**A, B**) Tumoricidal activities of splenocytes and peritoneal macrophages to a mouse cancer cell line at different ratios of effector cells to target cells. Data are presented as mean ± SEM and statistical analysis is performed with one-way ANOVA following Duncan’s multiple range test. (**C, D**) Levels of IFN-γ and TNFα in the culture supernatant of splenocytes and in the serum. Data are presented as mean ± SEM and statistical analysis is effectuated with one-way ANOVA following Tukey’s test. (**E**) Messenger RNA levels of IFN-γ (*Ifng*) and transcription factor t-bet (*Tbx21*) in the lymph nodes and spleen. Data were normalized to β-actin (*Actb*) expression levels and presented as n-fold changes to control group by 2^-∆∆CT^ method. Data are depicted as mean ± SEM and statistical analysis is executed with one-way ANOVA following Tukey’s test. (**F**) Extracellular polysaccharides (EPS) induced TNFα production in the peritoneal macrophages, but cordycepin could not. Lipopolysaccharides (LPS) at 1 μg/ ml was used as a positive control. Total protein levels were used for correction the total cell numbers. Data are presented as mean ± SD and N.D. means not detection. (**G**) IFN-γ and TNFα inhibited proliferation of 4NAOC-1 cells after 96 hours treatment. Data are presented as percentage relative to vehicle control and statistical analysis is conducted with one-way ANOVA following Tukey’s test. (**H**) Cordycepin decreased PD-L1 and PD-L2 expression in 4NAOC-1 cells. Representative results from three independent tests are shown.

Tumoricidal activities of splenocytes and peritoneal macrophages were significantly enhanced in LCM and HCM groups, compared with 4NA group (Figure 7A and 7B). Moreover, levels of IFN-γ and TNFα in the culture supernatant of splenocytes and in the serum were significantly increased in LCM and HCM groups (Figure 7C and 7D). Furthermore, the mRNA levels of IFN-γ (*Ifng*) and transcription factor t-bet (*Tbx21*) in the CLN, MLN, and spleen tissues were significantly increased in LCM and HCM groups (Figure 7E). We also tested the activities of active components in CMP. As shown in Figure 7F, EPS significantly increased TNFα production in peritoneal macrophages, and cordycepin could not. IFN-γ and TNFα significantly reduced proliferation of 4NAOC-1 cells (Figure 7G). Furthermore, cordycepin markedly down-regulated the expression of PD-L1 in 4NAOC-1 cells, but not PD-L2 (Figure 7H). These results prove that CMP and its components can enhance anti-tumor immune activities.

## DISCUSSION

We show that CMP can significantly inhibit tumor growth and malignant transformation in a mouse model for oral cancer. Cordycepin, an active component in CMP, can inhibit cancer cell proliferation, mitosis, and EGFR signaling, and increase cell apoptosis. In accordance, CMP can significantly inhibit the levels of ki-67 and EGFR signaling in cancer tissues. IL-17A can stimulate cancer cell proliferation and IL-17A levels in cancer tissues can be inhibited by cordycepin and CMP. Furthermore, cordycepin can suppress the expression of IL-17RA and its downstream signaling. In addition, CMP and cordycepin can significantly inhibit IL-17A production from lymphocytes *in vivo* and *in vitro*. Finally, CMP and its ingredients can enhance tumoricidal activities with increase in IFN-γ and TNFα, and decrease in PD-L1 expression.

EGFR is a membrane bound tyrosine kinase receptor, which phosphorylates MEK/Erk, STAT3, and mTOR signal molecules for regulation of cell survival and mitosis in many types of malignancy, including oral cancer [30]. It is highly expressed in head and neck SCC (HNSCC) [31], and its levels are adversely correlated with overall and disease-free survival, and positively with local relapse rate [32, 33]. Consequently, inhibition of EGFR expression and its signaling are a promising therapeutic strategy for oral cancer patients. Anti-EGFR monoclonal antibody, cetuximab has been approved for treatment of oral cancer. Our results show CMP can significantly inhibit tumor growth and malignant transformation in the model. In addition, CMP and cordycepin can significantly inhibit p-EGFR expression and its downstream signaling molecules p-Erk1/2, p-STAT3, and p-p70 S6K.

We found IL-17A was intensely expressed in SCC tissues from 4NA group. This cytokine was also highly expressed in many types of human cancer, including HNSCC [6, 34, 35], and its levels were positively associated with pathological grades and lymph node invasiveness [36]. IL-17A was reported to involve in many tumorigenic processes, including proliferation [8, 9, 37, 38], migration [38], invasion [39], angiogenesis [5], and malignant transformation [3]. More importantly, IL-17A levels were negatively correlated with disease-free survival rates in cancer patients, including HNSCC [6, 34, 35]. We showed CMP can inhibit IL-17A expression in SCC tissues. In addition, cordycepin can inhibit IL-17A stimulated proliferation in 4NAOC-1 cells, and inhibit the expression of IL-17RA and its signaling molecules, including p-Erk1/2, p-STAT3, and p-p70 S6K. In summary, these findings prove that CMP and cordycepin can inhibit tumor growth in which IL-17A plays a vital role for cancer cell proliferation.

IL-17A can be produced by lymphocytes, neutrophils, and mast cells [35], and lymphocytes are considered a major source. Here, we showed that cordycepin can inhibit production of IL-17A and IL-17A-expressing CD3^+^ CD4^+^ cells in a dose dependent manner. In addition, cordycepin can markedly inhibit IL-17RA and p-STAT3 and enhance p-STAT1 expression, and these factors are involved in the production of IL-17A and differentiation of IL-17A-expressing cells [4, 40, 41]. Anti-tumor agents, like resveratrol [42] and ursolic acid [43], were also reported to inhibit IL-17A production. Moreover, levels of IL-17A in the culture supernatant of CLN, MLN, and splenocytes and in the serum were significantly decreased in mice with CMP treatment. In summary, CMP and cordycepin are effective in inhibiting IL-17A production.

Tumoricidal activity is suppressed in many patients with HNSCC [28], and can be served as a predictor for prognosis [44]. Tumoricidal activity is dependent on cytokines, like IFN-γ and TNFα [12], which can activate Fas and caspase expression to induce apoptosis and cell death [13, 45]. In addition, transcription factor t-bet plays a pivotal role in the differentiation Th1 and Tc1 cells, which help to kill cancer cells [46]. Our results show that CMP can significantly enhance tumoricidal activity and the levels of IFN-γ and TNFα. CMP can also increase mRNA transcripts for *Ifng* and *Tbx21* in the lymph nodes and spleen. We showed IFN-γ and TNFα can induce cancer cell death after 96 hours’ incubation. One ingredient in CMP, EPS can significantly increase TNFα production in peritoneal macrophages.

PD-L1 plays as an immune checkpoint inhibitor, which is highly expressed in many types of cells, including cancer cells, and its levels can be inhibited by a mTOR pathway inhibitor, rapamycin [47]. Moreover, levels of PD-L1 were positively correlated with overall survival rate in patients with advanced HNSCC, who receiving anti-PD-1 therapy [48]. Anti-PD-L1 can increase IFN-γ and TNFα production in human natural killer T cells [49]. Our results show that cordycepin can inhibit PD-L1 expression in cancer cells, and another ingredient of CMP, EPS can significantly increase TNFα production in peritoneal macrophages. In summary, CMP and its ingredients can enhance anti-tumor activities.

The doses of CMP in the study are 500 mg/kg/day or 1500 mg/kg/day, equivalent to cordycepin at 15 μg/kg/day or 45 μg/kg/day, respectively. These doses can be translated to 25 or 75 ml/ day of 10-fold concentrated CMP for an 80 kg man.

We need to acknowledge some limitations in the study. First, we used MBT-2 cells as the target cells for assays of tumoricidal activity. The optimal target cells should be 4NAOC-1 cells. However, we had not yet established the cell line at the time of experiment. Despite MBT-2 cells are derived from mouse bladder cancer, they also express high levels of CK14, like 4NAOC-1 cells. Another limitation is that we did not have control mice feeding with blank culture medium. Blank medium is unlikely to have any anti-tumor effects.

## MATERIALS AND METHODS

### CM preparation (CMP)

The strain of *Cordyceps militaris* (CM) used in the study was identified and established by Dr. Tai-Hao Hsu (one of the correspondents), and deposited at the Bioresources Collection and Research Center, Food Industry Research and Development Institute, Hsinchu, Taiwan. It was maintained on potato-dextrose-agar (PDA) plates at 25°C. When mycelia grew into confluence, agar was cut into small pieces (1 cm^2^), and 15 pieces were seeded to a new liquid culture medium, consisting of 40 g/L sucrose, 10 g/L yeast extract, 0.5 g/L KH_2_PO_4_ and 0.5 g/L MgSO_4_·7H_2_O. A seed culture is defined as the submerged fermented culture at 25°C with rotation (100 rpm) in a shaker for 7 days. Subsequently, an inoculum (5%, v/v) derived from the seed culture was further inoculated into new culture medium. After another 7 days of cultivation (the final concentrations of residual sucrose and residual nitrogen in the cultured broth were less than 0.05%), the whole submerged fermented cultured broth was passed through a filter paper (Advantec No. 1, Toyo Roshi Kaisha Ltd., Tokyo, Japan) to remove mycelia. The filtered broth was further centrifuged at 6000 g for 30 minutes to remove precipitation. The product was lyophilized, and we used the dried material to make CMP used in this study. We used a HPLC system (RI-2031 Plus, JASCO Corp., Tokyo, Japan) to analyze CMP and the phenol-sulfuric acid assay to measure the levels of extracellular polysaccharides (EPS) [19, 20] in CMP.

### Induction of oral cancer and intervention with CMP

Male C57BL/6JNarl mice (4-week-old) were purchased from National Laboratory Animal Center (Taipei, Taiwan), housed in the animal facility of the Da-Yeh University, and accommodated for 2 weeks before the experiment. All the experimental protocols were approved by the Institutional Animal Care and Use Committee (IACUC) at the university and were accorded with the ARRIVE guidelines (Animal Research: Reporting of *In Vivo* Experiments). Mice took diet and water *ad libitum*. They were randomly divided into negative control group (receiving water), positive control group (receiving 4-nitroquinolin-1-oxide and arecoline, 4NA), and low dose (LCM) and high dose (HCM) of CMP groups. Mice received 500 mg/kg/day CMP in the LCM group and 1500 mg/kg/day in the HCM group via drinking water. The protocol was illustrated in Figure 1A. For induction of oral cancer, 4-nitroquinolin-1-oxide (4-NQO) (Sigma-Aldrich, MO, USA) was dissolved in propylene glycol and arecoline hydrobromide (Acros Organics, NJ, USA) was dissolved in pure water, respectively, to obtain stock solutions at 5 mg/ml, and stored at 4°C until use. After acclimatization for 2 weeks, mice were fed with either water or 4-NQO (100 μg/ml) and arecoline (500 μg/ml) in drinking water for 8 weeks. From the 9^th^ week, mice received either sterile water (Control and 4NA groups) or CMP-containing water (LCM and HCM groups) for the following 20 weeks.

### Observation and histopathological examination

After 8 weeks of exposure to carcinogens, mice were raised for another 20 weeks with or without CMP treatment. Starting from the 12th week, mice received gross examination of the full oral cavity under anesthesia with isoflurane (Panion & BF Biotech Inc., Taoyuan, Taiwan) bi-weekly, and were recorded by photography. Mice were sacrificed with carbon dioxide at the 28th week. Blood was collected by cardiac puncture. The tongue was cut, washed with normal saline, and photographed with a microscope (Olympus, Tokyo, Japan). Every lesion in the tongue was measured by a digital caliper (World Precision Instruments Inc., FL, USA). Tumor size was calculated by the following formula: tumor volume (mm^3^) = (length) * (width) * (width) * 0.5 [38]. Each tongue was longitudinally dissected into two parts. The tongue was fixed in formalin for 24 hours at room temperature. Subsequently, the tongue was washed with water, dehydrated with ethanol, and embedded in paraffin. For histopathological examination, paraffin blocks were cut into 4 μm sections, and stained with hematoxylin and eosin. The pathological grades of tongue lesions were categorized from mild to severe: hyperplasia, low-grade dysplasia (LGD), high-grade dysplasia (HGD), papilloma, squamous cell carcinoma (SCC) *in situ*, and invasive SCC [50]. Each pathological lesion was classified according to the most severe type of lesion. For example, if a large tumor comprised an invasive SCC lesion and a HGD lesion, it was graded as invasive SCC (Figure 1A). The multiplicity was defined as the number of lesions with the same pathological grade in one tongue.

### Immunohistochemical staining and quantification

Tissue blocks were cut into 4 μm thickness serial sections for immunohistochemical staining. These sections were deparaffinized, rehydrated, soaked with sodium citrate buffer for antigen retrieval, quenched with 3% H_2_O_2_ solution for depletion of endogenous peroxidase activity, and incubated with primary antibodies, including rabbit anti-ki-67 (#RM-9106, Thermo Fisher Scientific, CA, USA), rabbit anti-p-EGFR Y1086 (#ab40815, Abcam, Cambridge, UK), rabbit anti-p-STAT3 Tyr705 (# 04–1059, Millipore, CA, USA), and rabbit anti-IL-17A antibodies (#ab91649, Abcam, Cambridge, UK) overnight at 4°C. Subsequently, sections were incubated with goat anti-rabbit IgG HRP detection kit (GBI Labs., WA, USA), and developed with DAB Quanto substrate (Lab Vision Co., CA, USA). Finally, they were observed under an optical microscope (Olympus, Tokyo, Japan), and photographed by a software program (BestScope, Beijing, China). The expression levels of ki-67, p-EGFR, p-STAT3, and IL-17A in SCC tissues were quantified by Image-Pro Plus 6.0 software program (Media Cybernetics Inc., MD, USA). Data were presented as the percentage of the area with DAB staining over the cancer tissues area.

### Assays for tumoricidal activities

Splenocytes were suspended in RPMI 1640 medium, and co-cultured with MBT-2 mouse bladder tumor cells at different ratios for 20 hours. MBT-2 cells were pre-labeled with CFSE (Carboxyfluorescein succinimidyl ester, Molecular Probes, CA, USA) following the manufacturer’s instructions. Subsequently, all the cells were harvested, washed, stained with 7-AAD (7-aminoactinomycin D, Molecular Probes, CA, USA) for 20 minutes, and analyzed by a flow cytometry (Beckman Coulter Inc., CA, USA). The tumoricidal activity was defined as the percentage of CFSE and 7-AAD double positive MBT-2 cells over CFSE single positive MBT-2 cells. Either only MBT-2 tumor cells or only splenocytes were used as controls in each test. In the other experiment, peritoneal macrophages were obtained by washing the abdomen with DMEM medium. The lavage was collected in sterile tubes, treated with RBC lysis buffer, and cultured in 24-well plates at 1.5 × 10^6^ cells/ well. After overnight incubation, unattached cells were removed. On average, two thirds of seeding cells were peritoneal macrophages (1.0 × 10^6^ cells/ well). Subsequently, we added the CFSE-labeled MBT-2 cells at different effector/ tumor cell ratios for another 20 hours. All the cells were harvested, washed, stained with 7-AAD, and analyzed by flow cytometry. The tumoricidal activity was defined as mentioned before. Either only MBT-2 tumor cells or only macrophages were used as controls in each test.

### Establishment of 4NAOC-1 cell line and related assays

A tongue tumor from 4NA group was dissected, washed with antibiotics-containing PBS, digested with 0.1% collagenase I and IV for 3 hours at 37°C with gentle mechanical movement, removed RBC, and cultured in a 3-cm plate for 1.5 months with medium refreshment every 3–4 days. The completed culture medium was composed of DMEM/ F-12 (GE Healthcare Hyclone, UT, USA), antibiotics (Lonza Group Ltd., Basel, Switzerland), 20 ng/ml recombinant mouse epithelium growth factor and basic fibroblast growth factor (Prospec, Ness-Ziona, Israel), 10 μg/ml recombinant human insulin (Calbiochem, CA, USA), 1 μM dexamethasone (Calbiochem, CA, USA), and 10% fetal bovine serum (GE Healthcare Hyclone, UT, USA). A tongue tumor was resected from a mouse of the 4NA group, grinded, and cultured in the completed medium for four months in 2016. In the period, these cells were treated with 0.01% to 0.05% trypsin solution to remove fibroblasts from the primary culture in accordance with a published paper [51]. In the end, we obtained cells which were positive with flow cytometry for cytokeratin 14 (#ab181595, Abcam, Cambridge, UK) in more than 95% of cells. Cytokeratin 14 is present in oral epithelial cells, but not in fibroblasts. The final date of re-authentication was done in Jan. 2017 with the same method mentioned above.

The bioactive compounds used for *in vitro* experiments were purchased from Sigma-Aldrich, except for EPS, which was purified in our laboratory following a published method [20]. The survival rate and proliferation rate of 4NAOC-1 cells was effectuated with alamarBlue^®^ cell viability assay protocol (Thermo Fisher Scientific Inc., CA, USA). The colony formation assay was done by seeding 1 × 10^4^ cells/ well in 6-well culture plates with or without treatment for 5 days, then cells were fixed, stained with crystal violet, counted, and taken pictures. The soft agar assay was performed with the lower agar layer (0.5%) and the upper agar layer (0.3%) with or without treatment for 4 weeks, then cells were stained with crystal violet, counted, and photographed. For apoptosis and cell cycle analysis, cells were treated with CMP or cordycepin at indicated concentrations for three days; subsequently, cells were collected, washed with PBS, stained with annexin v/ 7-AAD (BD Biosciences, CA, USA) or propidium iodide (PI, BD Biosciences, CA, USA), and analyzed by a flow cytometer. For IL-17A, IFN-γ, and TNFα stimulation, cells were seeded in the culture plate for 24 hours with serum starvation, then cells were stimulated with recombinant mouse IL-17A, IFN-γ, and TNFα (Biolegend Inc., CA, USA) for the indicated time periods. For 5-Bromo-2´-Deoxyuridine (BrdU) proliferation assay, cells were treated with IL-17A and/ or cordycepin for consecutive 3 days, and BrdU was added at the last 24 hours (final concentration 10 μM), then cells were washed with PBS, fixed, permeabilized, and stained with anti-BrdU monoclonal antibody (Abcam, Cambridge, UK).

### Western blotting

Equivalent total protein lysates were loaded in each lane, run on SDS-PAGE, transferred to PVDF membranes, blocked with 5% skim milk, incubated with primary antibodies overnight at 4°C, including anti-p-EGFR Y1092(#ab40815, Abcam, Cambridge, UK), EGFR (#ab52894, Abcam, Cambridge, UK), β-catenin (#ab32572, Abcam, Cambridge, UK), cyclin B1 (#4135, Cell Signaling Technology Inc., MA, USA), p-p70 S6K Thr421/ Ser424 (#9204, Cell Signaling Technology Inc., MA, USA), p70 S6K (#2708, Cell Signaling Technology Inc., MA, USA), p-Erk1/2 Thr202/ Tyr204 (#9101, Cell Signaling Technology Inc., MA, USA), Erk1/2 (#4696, Cell Signaling Technology Inc., MA, USA), p-STAT3 Tyr705 (#9145, Cell Signaling Technology Inc., MA, USA), STAT3 (#569388, Calbiochem, CA, USA), p-STAT1 Y701 (#ab29045, Cell Signaling Technology Inc., MA, USA), STAT1 (#06–501, EMD Millipore Corporation, CA, USA), GAPDH (#ab181602, Abcam, Cambridge, UK), and β-actin (#A5441, Sigma-Aldrich, MO, USA), and observed under a chemiluminescence system.

### Flow cytometric assays for membrane proteins

After cordycepin treatment, 4NAOC-1 cells were washed with PBS, fixed, permeabilized with 0.1% triton x-100, blocked with anti-CD16/32 monoclonal antibody (eBioscience, CA, USA), and stained with IL-17RA (eBioscience, CA, USA), PD-L1 (Biolegend, CA, USA), and PD-L2 (Abcam, Cambridge, UK) monoclonal antibodies for 2 hours at room temperature. Subsequently, cells were analyzed with flow cytometry (Beckman Coulter FC500, CA, USA), and results were plotted with Flowing Software 2.5.1 (Cell Imaging Core, Turku Centre for Biotechnology).

### *Ex vivo* mononuclear cell stimulation

At sacrifice, the cervical lymph nodes (CLN), mesenteric lymph nodes (MLN), and the spleen were immediately dissected. Lymph node tissues were grinded by a homogenizer, treated with Ficoll-paque PLUS (GE Healthcare Bio-sciences AB, Sweden), and removed erythrocytes by a RBC lysis buffer (BD Biosciences, CA, USA). Recovered cells were suspended in a 10% FBS RPMI 1640 medium with 1% penicillin and streptomycin. Subsequently, these recovered cells (1.2 × 10^6^ cells/ ml) were stimulated with phorbol 12-myristate 13-acetate (PMA, Calbiochem) at 50 ng/ml and ionomycin at 500 ng/ml (Calbiochem) for 36 hours at 37°C. The culture supernatants were collected and stored at –80°C until use.

### ELISA for cytokines in the serum and culture supernatants

Before analysis, all samples were centrifuged to remove debris. Cytokine levels were measured by ELISA kits (eBioscience, CA, USA and R&D systems Inc., MN, USA) and all the procedures followed the manufacturers’ instructions.

### *In vitro* differentiation of IL-17A-expressing cells

Splenocytes from naïve B6 mice and cervical lymph node cells from tongue tumor-bearing mice were cultured with Th17 promoting agents for 3 days. Cells were pre-treated with cordycepin or other ingredients for 3 hours, following co-cultured with Th17 promoting agents for 3 days. The Th17 promoting agents were composed of anti-CD3 mAbs (1 μg/ml, clone 145–2C11), anti-CD28 mAbs (1 μg/ml, clone 37.51), anti-IFN-γ mAbs (1 μg/ml, clone XMG1.2), anti-IL-4 mAbs (1 μg/ml, clone 11B11), rIL-6 (50 ng/ml, Biolegend), rIL-23 p19 (5 ng/ml, Biolegend), and rTGF-β (1 ng/ml, Biolegend). After 3 days, cells and supernatants were collected. Cells were re-stimulated with PMA (50 ng/ml) and ionomycin (500 ng/ml) for 8 hours with monensin and brefeldin A. Subsequently, cells were harvested, permeabilized with Cytofix/Cytoperm solution (BD Biosciences, CA, USA), blocked with anti-CD16/32 mAbs (eBioscience, CA, USA), and stained with anti-CD3 (clone 145–2C11), anti-CD4 (clone RM4-5), and anti-IL-17A (clone TC11-18H10) mAbs for detection of IL-17A-expressing cells by flow cytometry. Levels of IL-17A and LDH (Takara Bio Inc., Kyoto, Japan) in the culture supernatants were analyzed with ELISA kits.

### *Ex vivo* TNFα stimulation

Peritoneal macrophages of naïve B6 mice were treated with cordycepin or EPS for 24 hours at 1 × 10^6^ cells/ well. The cultured supernatants were collected, and analyzed levels of TNFα with an ELISA kit (eBioscience, CA, USA). The attached macrophages were lysed with lysis buffer, and measured total amounts of protein by BCA method.

### Measurement of mRNA transcripts

Total RNA was isolated from lymph node cells and splenocytes with a commercial RNA purification kit (Zymo Research Corporation, MA, USA). Total RNA was reversed by a MMLV transcriptase (Applied Biosystems, CA, USA) to form the cDNA. Levels of mRNA transcript were determined with a SYBG green real-time PCR master mix kit (Toyobo Co., LTD, Osaka, Japan) on ABI 7300 real-time PCR system (Applied Biosystems, CA, USA). No template control was used as a negative control to avoid any contamination. The expression level of each target gene was normalized to β-actin (ΔCt) and plotted as n-fold changes to control group (2-ΔΔCt).

### Statistical analysis

We used one-way ANOVA following Tukey’s test (GraphPad Software, CA, USA) for all the experimental data, except for the tumoricidal activities, which were analyzed by one-way ANOVA following Duncan’s multiple range test (SPSS v20, IBM Corporation, NY, USA). The incidence was effectuated with Fisher’s exact test by SPSS software. *P* value smaller than 0.05 is considered statistically significant.

## CONCLUSIONS

CMP and its ingredient cordycepin can inhibit carcinogenesis in a murine model for oral cancer. The anti-tumor mechanisms depend on inhibition of EGFR- and IL-17RA-signaling and enhancement of anti-tumor immune responses. Further clinical studies are needed to prove the therapeutic effects of CMP in cancer patients, particularly in whom EGFR and IL-17RA play a pivotal role.

## SUPPLEMENTARY MATERIALS FIGURE


