# Supplementary material for: Cordycepin and a preparation from Cordyceps militaris inhibit malignant transformation and proliferation by decreasing EGFR and IL-17RA signaling in a murine oral cancer model
Source: Oncotarget. 2017 Oct 4;8(55):93712–28. doi: 10.18632/oncotarget.21477 (PMC5706830; doi:10.18632/oncotarget.21477)
Supplement: Supplementary file 1 [file oncotarget-08-93712-s001.pdf]

Cordycepin and a preparation from *Cordyceps militaris* inhibit malignant transformation and proliferation by decreasing EGFR and IL-17RA signaling in a murine oral cancer model

SUPPLEMENTARY MATERIALS

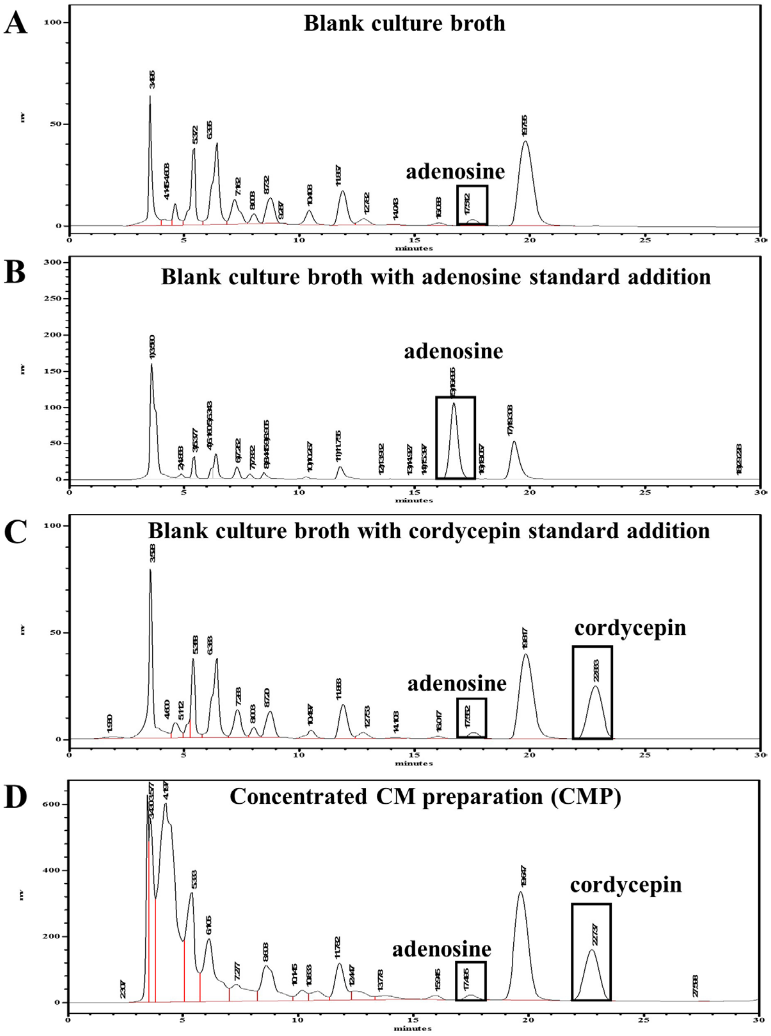

E

Content	Concentration
Cordycepin	5.23 ± 0.01 µg/ml
Adenosine	0.38 ± 0.06 µg/ml
D-mannitol	16.38 ± 0.54 µg/ml
Extracellular polysaccharides (EPS)	18.43 ± 8.80 mg/ml

Supplementary Figure 1: HPLC and *in vitro* analysis of CMP. (**A**) Blank culture broth. (**B**) Blank culture broth with adenosine supplement. (**C**) Blank culture broth with cordycepin supplement. (**D**) Concentrated CMP. Detection of ingredients in the CMP was performed with a C18 column at 25°C with 15% methanol as mobile phase and UV detector at the wavelength of 254 nm. The flow rate was 0.7 ml/ min and the sample volume was 20 µl. (**E**) Concentrations of major bioactive ingredients in the CMP. Data were presented as mean ± SD and three different batches of CMP were determined. CMP: Cordyceps militaris preparation.
